# Evaluation of antioxidant and antibacterial interactions between resveratrol and eugenol in carboxymethyl cellulose biodegradable film

**DOI:** 10.1002/fsn3.2656

**Published:** 2021-11-15

**Authors:** Majid Aminzare, Roya Moniri, Hassan Hassanzad Azar, Mohammad Reza Mehrasbi

**Affiliations:** ^1^ Department of Food Safety and Hygiene School of Public Health Zanjan University of Medical Sciences Zanjan Iran

**Keywords:** antimicrobial, antioxidant, carboxymethyl cellulose, eugenol, resveratrol

## Abstract

The aim of present study was to compare the in vitro antioxidant and antibacterial properties of carboxymethyl cellulose (CMC) films containing resveratrol (RES) and eugenol (EUG), alone and in combination, and to calculate the dose interactions between them. At first, the total phenolic content of CMC films was evaluated. Then, their antioxidant and antibacterial effects of films were determined using DPPH, reducing power, disk diffusion, and broth dilution methods. Finally, concentrations of RES and EUG which showed better results in the CMC films were added in combination forms to calculate their antioxidant and antibacterial interactions. The results showed that addition of RES and/or EUG to CMC films increased the total phenolic content, free radicals scavenging activity, reducing power, and antibacterial activities of the films (*p* ≤ .05). Gram‐positive bacteria were more susceptible than Gram‐negatives. In addition, the combined use of RES and EUG in CMC films had synergistic antioxidant and antagonistic antibacterial effects. The best results belonged to the film containing RES (8 µg/ml) + EUG (8 mg/ml) (*p* ≤ .05). Considering the results of the present research, we can utilize CMC biodegradable film containing RES and EUG as a natural active packaging in food industry.

## Graphical abstract

The aim of present study was to compare the in vitro antioxidant and antibacterial properties of carboxymethyl cellulose (CMC) films containing resveratrol (RES) and eugenol (EUG), alone and in combination, and to calculate the dose interactions between them. The combined use of RES and EUG in CMC films had synergistic antioxidant and antagonistic antibacterial effects.

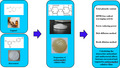

## 1 INTRODUCTION

Food‐borne pathogens are considered as the most salient public health problems and cause illness and death in a significant number of people every year. Despite recent advances in food safety science, foodborne diseases are still a major concern (Newell et al., 2010). Among microbial pathogens, bacteria are the most common. Because inappropriate actions for food preparation and distribution cause contamination, survival and growth of pathogenic bacteria. Some of mentioned bacterial species include *Salmonella enteritidis*, *Escherichia coli*, *Staphylococcus aureus,* and *Listeria monocytogenes* (Greig & Ravel, 2009; Hobbs et al., 2017; Newell et al., 2010). On the other hand, lipid oxidation is another matter of concern that endangers consumer safety and, more broadly, public health. Because in addition to economic losses, it reduces the sensory and nutritional properties of the product, threatens the health of consumers, and causes cancer and cardiovascular disease (Grootveld et al., 2020; Nieva‐Echevarría et al., 2020).

Food packaging is one of the most important processes for maintaining the microbial and chemical quality and safety of products (Han, 2005). Nowadays, synthetic polymers are widely used in food packaging industry due to their desirable characteristics such as stability, flexibility, and ease of production (Yadav et al., 2018). However, synthetic polymers can cause problems such as adverse environmental effects, the release of toxic gases, and contribution to global warming. In addition, the transfer of chemical compounds such as monomers and solvent residues, to food is another problem of synthetic polymers. Environmental and consumer health concerns have led to an increase in the replacement of biodegradable polymers with synthetic polymers (Mignon et al., 2019). Biodegradable polymers such as edible films can be considered as a suitable barrier against moisture and gases (oxygen and carbon dioxide) as well as good carriers for antimicrobial and antioxidant compounds as active packaging (Hasheminya et al., 2019). Carboxymethyl cellulose (CMC) is one of the most widely used polymers in the food packaging industry due to its hydrophilic properties, optimal film ductility, biological compatibility, gas transfer inhibition, and internal stability of the network structure (Roy & Rhim, 2020). This biodegradable polysaccharide is non‐toxic and shows good functional characteristics in the formation of active packaging (Raeisi et al., 2012). Previous studies have shown that CMC film and coating are good carriers for several bioactive agents such as natural antimicrobial and antioxidant compounds (Dashipoor et al., 2014; Dashipour et al., 2015; Panahirad et al., 2021; Raeisi et al., 2015). According to Panahirad et al. (2021), CMC‐active coatings could reserve active ingredients in their structure and gradually release them to the surface of fruits and vegetables to improve the performance of the active ingredients. As a result, the quality and shelf life of the product will be promoted (Panahirad et al., 2021).

The use of antimicrobial and antioxidant compounds in food products is a good solution to maintain the safety of consumers. Despite the widespread use of synthetic antimicrobial and antioxidant compounds in food industry, the adverse effects of these compounds on human health are considered as a serious public health concern. In this respect, comprehensive researches have been performed to replace them with natural compounds (Davidson et al., 2014; Shahidi & Ambigaipalan, 2015).

Resveratrol (RES) is a stilbene polyphenolic compound which exists in grapes, peanuts, red wine, and mulberries. Several studies have been conducted and reported antimicrobial, antioxidant, and potential health benefits of resveratrol (Arcanjo et al., 2019; Ma et al., 2018; Oh & Shahidi, 2018).

Eugenol (EUG) is a natural and aromatic compound that is generally extracted from cloves, bay laurel, and cinnamon bark. This phenolic compound has been interested as an additive applied in the food, agricultural, pharmaceutical, and cosmetic industries, due to its low‐cost, and desirable antimicrobial and antioxidant activities (Zheng et al., 2019).

Previously, several studies have been performed on the antimicrobial and antioxidant potential of biodegradable films containing resveratrol and eugenol alone (Busolo & Lagaron, 2015; Ma et al., 2018; Pastor et al., 2013; Suppakul, 2016; Zheng et al., 2019). But according to best of our knowledge, no comparative studies have been conducted on the antimicrobial and antioxidant potential of resveratrol and eugenol in biodegradable films yet. Therefore, the objective of the present study was to compare the antibacterial and antioxidant properties of CMC biodegradable films containing RES and EUG, alone and in combination, and to calculate the dose interactions between them using an in vitro model.

## 2 MATERIALS AND METHODS

### 2.1 Materials

Materials were supplied from companies as follow: butylated hydroxytoluene (BHT), calcium chloride, potassium phosphate buffer, potassium ferricyanide, trichloroacetic acid, ferric chloride, ethanol, methanol, sodium carbonate, glycerol, Folin–Ciocalteu reagent, 1 1‐diphenyl‐2‐picrylhydrazyl (DPPH), CMC powder, resveratrol powder, and Gallic acid were purchased from Sigma (Sigma‐Aldrich). Also, brain heart infusion (BHI) agar, BHI broth, and nutrient agar media were purchased from Merck (Merck). Lyophilized vials of *Staphylococcus aureus* (ATCC 25923), *Salmonella enteritidis* (ATCC 14028), *Listeria monocytogenes* (ATCC 13932), and *Escherichia coli* (ATCC 15224) were bought from microbial collection of the Iranian Research Organization for Science and Technology (IROST).

### 2.2 Preparing CMC films

In order to preparation of CMC films containing RES and EUG, one gram of CMC powder and 0.01 g calcium chloride were dissolved in 100 ml of sterile distilled water. The film solution was then stirred using a magnetic stirrer at 70°C and 1200 rpm for 45 min to completely dissolve the CMC and to obtain a uniform solution. Then, 0.5 ml of glycerol (50% v/w based on CMC weight) was added to the suspension as a plasticizer and was mixed at 70°C for 10 min. Subsequently, the obtained solution was cooled to 55°C and several concentrations of RES (1, 2, 4, and 8 μg/ml) and EUG (1, 2, 4, and 8 mg/ml) were separately added to the film‐forming solution, alongside Tween 80 (as emulsifier and in similar concentrations). Concentrations of RES and EUG which showed better antibacterial and antioxidant effects were also incorporated into CMC film in combination form to evaluate the dose interactions between them. Then, film‐forming solutions were homogenized with ultra‐turrax homogenizer (IKA T10 basic) at 13,500 rpm for 3 min at room temperature. CMC solution without addition of RES and EUG was considered as the control group. Then, 25 ml of the film‐forming solutions was poured on Teflon plates (ϕ = 10 cm) and placed in the oven to dry for 30 h at 37°C. The dried films were then separated from the plates and stored in a desiccator at 25°C and 53% relative humidity until testing. Prior to antibacterial testing, the films were exposed to ultraviolet light for 2 min under the laminar hood in order to eliminate possible contaminants (Dashipour et al., 2015).

### 2.3 Total phenolic content of CMC films containing RES and EUG

Folin–Ciocalteu method was used to measure the phenolic compounds of CMC films containing different concentrations of RES and EUG. For this purpose, 25 mg of each film was placed in 3 ml of distilled water and it was homogenized for 5 min. Then, 0.1 ml of the film extract was mixed with 7 ml of distilled water and 0.5 ml of Folin–Ciocalteu reagent and kept at room temperature for 8 min. Consequently, 1.5 ml of sodium carbonate (2% w/v) and distilled water were added to obtain the final volume of 10 ml. Afterward, the obtained mixture was kept in darkness at the room temperature for 2 h and the absorbance was measured at 765 nm by a spectrophotometer (HACH, DR 5000). The calibration curve was drawn using gallic acid at specific concentrations, and the results were determined using the following equation:

T=CV/M.

where T is total phenolic content (mg GAE/g film), C is the gallic acid concentration obtained from the standard curve (mg/ml), V is the volume of film extract (ml), and M is the weight of dried film (g). The experiment was carried out in triplicate (Moradi et al., 2011).

### 2.4 Evaluation of antioxidant activities of CMC films containing RES and EUG

#### 2.4.1 DPPH free radical scavenging activity

25 mg of each film sample was dissolved in 3 ml of distilled water, and then, 2.8 ml of each film extract was added with 0.2 ml of 1 mM DPPH methanolic solutions. The absorbance was measured at 517 nm by a spectrophotometer after incubation for 30 min at room temperature. Films which contain 1 mg/ml BHT and film without any antioxidant compound were considered as positive and negative controls, respectively. The percentage of inhibition was measured by the following equation:

$$\text{DPPH radical scavenging activity}\;\left(\%\right)=\left({\text{Abs}}_{\text{DPPH}}‐{\text{Abs}}_{\text{sample}}\right)/{\text{Abs}}_{\text{DPPH}}\times 100.$$

where Abs_DPPH_ is the absorption of DPPH methanolic solution and Abs_sample_ is the absorption of CMC films (Hashemi et al., 2019).

#### 2.4.2 Ferric (Fe^3+^) reducing power

At first, 10 mg of each film was added to the test tubes containing 1.25 ml of potassium phosphate buffer (pH 6.6, 0.2 M) and 1.25 ml of potassium ferricyanide (1%), and it was kept at 50°C for 20 min. Afterward, 0.5 ml of trichloroacetic acid (10%) was added to the tubes and centrifuged at 3000 rpm for 10 min. Finally, 1.25 ml of the supernatant was transferred to the tubes containing 1.25 ml of potassium phosphate buffer and 0.25 ml of ferric chloride. After 10 min incubation, the absorbance of test tubes was measured at 700 nm. The higher adsorption rate of the final mixture, the greater the reducing power of the sample (Jridi et al., 2019).

#### 2.4.3 Calculating the antioxidant interactions between RES and EUG in CMC films

The antioxidant interactions between RES and EUG in CMC films which were determined by DPPH and reducing power tests were calculated using the combination index (C.I). For this purpose, the following formulas were used:

$$C\text{.I}=\frac{{\text{DPPH}\;}_{\text{ab}}/2}{{\text{DPPH}\;}_{a}}+\frac{{\text{DPPH}}_{\text{ab}}/2}{{\text{DPPH}\;}_{b}},$$

$$C\text{.I}=\frac{R{\text{.P}\;}_{\text{ab}}/2}{R{\text{.P}}_{a}}+\frac{R{\text{.P}}_{\text{ab}}/2}{R{\text{.P}}_{a}}.$$

where DPPH_ab_ and R.P_ab_ are the obtained results from the combined use of RES and EUG in CMC films, and DPPH_a_, DPPH_b_, R.P_a_, and R.P_b_ are the values obtained from the use of RES and EUG alone in CMC films. C.I values equal, smaller or greater than 1, indicate an additive, antagonistic, or synergistic effects, respectively (Hashemi et al., 2019).

### 2.5 Evaluating of antibacterial activities of CMC films containing RES and EUG

#### 2.5.1 Preparation of studied bacterial strains

Antibacterial effects of CMC films were studied against two gram‐positive bacterial strains including *S. aureus* and *L. monocytogenes*, and two gram‐negative bacterial strains including *E. coli* and *S*. *enteritidis*. First of all, stock bacteria (vials containing 20% w/v glycerol in BHI broth and kept at −20°C) were inoculated in 15 ml BHI broth and incubated at 37°C for 24 h at 150 rpm (repeated at least twice). Afterward, inoculums were centrifuged three times at 6000 rpm for 5 min to be separated from BHI broth. During the last 2 centrifugation steps, the supernatant was eliminated and physiological saline was added. In order to prepare and count bacterial inoculations, optical density (OD) method was used. Various dilutions were prepared from bacterial cultures, and their absorbance was read at 600 nm using a spectrophotometer to adjust to 0.5 McFarland standard turbidity (~1–2 × 10^8^ CFU/ml). Afterward, bacterial suspensions were diluted to achieve the desired concentration for each test. In order to confirm the results, bacterial counting was carried out in BHI agar at 37°C for 24 h (Abdollahzadeh et al., 2014).

#### 2.5.2 Disk diffusion method

In this test, 100 µl of each studied bacterial suspension (~1–2 × 10^6^ CFU/ml) was cultured in BHI agar medium. Consequently, disks with a diameter of 6 mm were prepared from CMC films containing different concentrations of RES and EUG (alone and in combination) and placed on the culture medium. Disks which contain chloramphenicol (30 μg/disk) and CMC films without any antimicrobials were used as positive and negative controls, respectively. Then, the plates were incubated at 37°C for 24 h and antimicrobial activity of CMC films was assessed by measuring the inhibition zone diameter by a caliper (Moghimi et al., 2017). Accordingly, the antibacterial activities of films were classified as follows (Lv et al., 2011):

12 mm ≥ inhibition zone: Weak antibacterial activity;

20 mm > inhibition zone > 12 mm: Moderate antibacterial activity;

20 mm ≤ Inhibition zone: Strong antimicrobial activity.

##### Calculating the antibacterial interactions between RES and EUG in CMC films using disk diffusion method

To evaluate the antibacterial interactions between RES and EUG in CMC films, at first the inhibition zone of CMC films containing RES was statistically added to the inhibition zone of CMC films containing EUG and recorded as “expected inhibition zone”. Then, the inhibition zone of CMC films containing a combination of RES and EUG at similar concentrations was compared with them. If the expected inhibition zones are equal, smaller or greater than the inhibition zones of CMC films containing a combination of RES and EUG, indicate an additive, synergistic or antagonistic effects, respectively (Moussaoui & Alaoui, 2016).

#### 2.5.3 Broth dilution method

In this method, 25 mg of each CMC films was transferred to test tubes containing 10 ml of bacterial suspension (BHI broth containing ~1–2 × 10^5^ CFU/ml bacteria). Afterward, the tubes were placed in shaking incubator at 37°C for 24 h. A test tube without film and a test tube without bacteria were used as positive and negative controls, respectively. Then, 0.1 ml of each tube was used to prepare serial dilutions that were spread on nutrient agar plates. The plates were incubated at 37°C for 24 h, and the colonies were counted and calculated using a dilution factor (Sugumar et al., 2015). In order to perform the next calculations, positive control was counted immediately after preparing the suspension and 24 h after incubation.

#### 2.5.4 Calculating the bacterial reduction index

In order to calculate the bacterial reduction index of CMC films containing different concentrations of RES and EUG, the international standard method (ISO, 22196:^2007^) was used with some modification (22196, 2007). For this purpose, the number of countable colonies obtained by the broth dilution method was used in the following formula to quantify the bacterial reduction index:

$$B\text{.R}=\;\left[\log\;\left(B/A\right)\;‐\;\log\;\left(C/A\right)\right]\;=\;\log\;\left(B/C\right).$$

where B.R is the bacterial reduction index; A is the mean of bacterial counts of the control sample immediately after preparing the suspension; B is the mean of bacterial counts of the control sample after 24 h (24‐h control); C is the mean of bacterial counts of the treated samples after 24 h.

Therefore, the antimicrobial performance of the films was determined as follows (Torres‐Giner et al., 2017):

0.5 > B.R: Nonsignificant antibacterial activity;

1 > B.R ≥ 0.5: Slight antibacterial activity;

3 > B.R ≥ 1: Significant antibacterial activity;

3 ≤ B.R: Strong antibacterial activity.

##### Calculating the percentage of bacterial growth inhibition

The percentage of bacterial growth inhibition CMC films containing various concentrations of RES and EUG was calculated using the following formula:

$$P\text{.I}\left(\%\right)\;=\;\left(\text{CON}‐\text{Treatment}/\text{CON}\right)\times 100.$$

where P.I is the percentage of bacterial growth inhibition; CON is the mean of bacterial counts of the control sample after 24 h (24‐h control); Treatment is the mean of bacterial counts of the treated samples after 24 h.

### 2.6 Statistical analysis

All tests were performed in triplicate. Statistical analysis of the data was performed using one‐way ANOVA with SPSS software (Version 18.0 for Windows; SPSS Inc.). Tukey test was also used to determine the significant difference between the means (*α* = .05). Pearson test was also used to determine the correlation between the data.

## 3 RESULTS AND DISCUSSION

### 3.1 Total phenolic content

Phenolic compounds, including terpenes and flavonoids, are responsible for the strong antimicrobial/antioxidant activities of some plant preservatives (Aziz & Karboune, 2018). The total phenolic compounds in the CMC films containing different concentrations of RES and/or EUG using Folin–Ciocalteu method was expressed as mg GAE/g film which was calculated by using an equation that was obtained from the standard graph (R² = 0.9992):

$${\text{Abs}}_{765}=0.0011\;\text{mg gallic acid}+0.0075.$$

**FIGURE 1:**
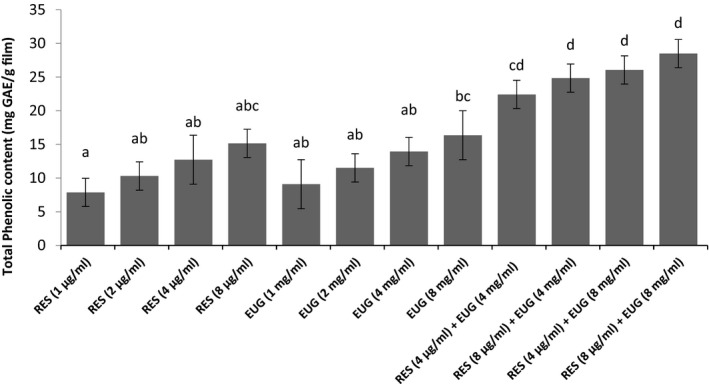
Total phenolic content of CMC films incorporated with RES and EUG (Mean ± SD). Values followed by different letters are significantly different according to Tukey's Multiple Range Test (*p* ≤ .05)

Based on the results (Figure 1), the amount of total phenolic compounds in CMC films containing different concentrations of RES and EUG was in the range of 7.88–15.15 and 9.09–16.36 mg GAE/g film, respectively. In this regard, Talón et al. (2019) have reported 3.24 mg GAE/g film total phenolic content in corn starch film containing 1.59% EUG (Talón et al., 2019). Also, according to the study conducted by Adhikari et al. (2018), the results showed that there was a positive correlation between the total phenolic content and the amount of RES in seeds and sprouts of Korean peanuts (Adhikari et al., 2018). In another study, the total phenolic content of sodium alginate film containing 0.25–4 μg/ml RES was 6.82–11.56 mg GAE/g film which was in accordance with the results of present study (Hashemi et al., 2019). Moreover, the results of present study showed that there was a significant increase in total phenolic content following an increase in RES and EUG concentrations in CMC films (*p* ≤ .05). In agreement with these results, the total phenolic content of gelatin and chitosan film‐forming solutions containing 1%–10% EUG was reported in the range of 2222.63–3491.97 and 2920.63–3580.97 of mg GAE/L of film‐forming solution, respectively (Baygar, 2019). Dashipoor et al. (2014) also reported that the total phenolic content of CMC films increased with increasing the concentration of clove essential oil (containing EUG as the main phenolic compound) (Dashipoor et al., 2014). Also, the phenolic content of the films containing the combination of RES and EUG was significantly higher than other films (*p* ≤ .05). The highest total phenolic content belonged to the film containing RES (8 µg/ml) + EUG (8 mg/ml) with the score of 28.48 mg GAE/g film which was significantly higher than alone use of similar concentrations of RES or EUG in CMC films (*p* ≤ .05).

### 3.2 Antioxidant activities of CMC films containing RES and/or EUG

#### 3.2.1 DPPH free radical scavenging activity

DPPH method is a comprehensive test for measuring the free radical scavenging ability of edible films containing natural antioxidants with plant origin. This method is very suitable for evaluating the free radical scavenging power due to its simplicity, speed, and independence from sample polarity (Salarbashi et al., 2014). The results of antioxidant activity of CMC films containing RES or EUG measured using DPPH free radical scavenging method are shown in Table 1. As can be seen, the films prepared without adding RES and EUG (control group) did not show any antioxidant activity and the antioxidant activities of treated films with different concentrations of RES and EUG were in the range of 61%–87% and 72%–87.67%, respectively. Also, the antioxidant properties of all films were significantly increased by increasing the concentrations of RES or EUG to CMC films (*p* ≤ .05). The highest antioxidant capacities between CMC films containing RES or EUG, belonged to the concentrations of 8 μg/ml RES and 8 mg/ml EUG (*p* ≤ .05). Also, the antioxidant capacity of CMC film containing 1 mg/ml BHT was 79%. Given the studied concentrations, it can conclude that the antioxidant capacity of the positive control is lower than the film containing RES and higher than the films containing EUG. In the study by Zheng et al. (2019), it was reported that the antioxidant capacity of chitosan–acorn starch films containing 1.2% EUG by DPPH method was about 86.77% which was in accordance with the results of the present study. In the aforementioned study, the antioxidant capacity of the films has been attributed to the reaction between the hydroxyl groups of EUG and free radicals and the formation of stabilized phenoxyl radicals. Film containing EUG is able to act as a strong donor of electrons or hydrogen atoms and subsequently turn purple color of DPPH free radicals to yellow (Zheng et al., 2019). In a study conducted by Busolo and Lagaron (2015), it was reported that polyethylene film enriched with 1000 ppm RES had antioxidant activity of about 80% based on the DPPH method (Busolo & Lagaron, 2015). In another study, chitosan and methylcellulose films containing RES showed favorable antioxidant activity and it was recommended that these films could be used as a packaging material for foods which are sensitive to autoxidation (Pastor et al., 2013). In the study by Hashemi et al. (2019), the DPPH free radical scavenging activity of sodium alginate film containing 4 µg/ml RES was 72.66%, which verifies the results of present study (Hashemi et al., 2019). Other researchers have proven that RES binds to metal ions and exhibits chelating activity, thus preventing the oxidation process by preventing the overproduction of hydroxyl radicals (Truong et al., 2018).

**TABLE 1:** DPPH scavenging activity of CMC films incorporated with RES or EUG (Mean ± SD)

Sample	Concentration	Scavenging activity (%)	Sample	Concentration	Scavenging activity (%)
RES (µg/ml)	0	—	EUG (mg/ml)	0	—
	1	61.00 ± 2.65^a^		1	72.00 ± 2.00^a^
	2	74.33 ± 2.08^b^		2	77.33 ± 1.53^b^
	4	79.33 ± 2.52^b^		4	82.33 ± 1.53^c^
	8	87.00 ± 1.00^c^		8	87.67 ± 1.53^d^
BHT (mg/ml)	1	79.00 ± 1.41^b^	BHT (mg/ml)	1	79.00 ± 1.41^bc^

Note

Values followed by different letters within the same columns are significantly different according to the Tukey's test (*p* ≤ .05).

#### 3.2.2 Ferric (Fe^3+^) reducing power

Since the antioxidant activity of films depends on diverse factors with various mechanisms of action, it is necessary to use more than one method to determine the in vitro antioxidant capacity of films (Pérez‐Jiménez et al., 2008). Therefore, in the present study, the antioxidant capacity of the films was also investigated by the reducing power method. In this method, the ability of the antioxidant compound to reduce Fe^3+^ to Fe^2+^ is measured. The formed Fe^2+^ can react with ferric chloride to form ferric ferrous complex that has an absorption maximum at 700 nm. The higher absorption rate indicated the greater reduction power of the samples (Woranuch & Yoksan, 2013). The results of antioxidant activity of CMC films incorporated with RES or EUG which were determined by reducing power assay are shown in Table 2. According to the results, the reducing power of the films containing different concentrations of RES and EUG was in the range of 0.21–0.59 and 0.22–1.41, respectively. Also, the reducing power of all films was significantly increased by increasing the concentrations of RES or EUG to CMC films (*p* ≤ .05). The highest reducing power among films containing RES was related to the concentration of 8 μg/ml and among the films which contained EUG was related to the concentration of 8 mg/ml (*p* ≤ .05). Additionally, the adsorption rate of films containing 1 mg/ml BHT was 0.7. Woranuch and Yoksan (2013) reported that the reducing power of film which was prepared from thermoplastic flour containing 0.7% EUG was 0.31 (Woranuch & Yoksan, 2013). It was proven that RES has a significant ability to reduce Fe^3+^ to Fe^2+^, and this ability increases with increasing the concentration of RES. Furthermore, it has been declared that RES has a greater reducing power than BHT. The final outcome of reducing reactions is to terminate the radical chain reactions (Gülçin, 2010).

**TABLE 2:** Reducing power of CMC films incorporated with RES or EUG (Mean ± SD)

Sample	Concentration	Absorbance at 700 nm	Sample	Concentration	Absorbance at 700 nm
RES (µg/ml)	0	0.01 ± 0.00^a^	EUG (mg/ml)	0	0.01 ± 0.00^a^
	1	0.21 ± 0.01^b^		1	0.22 ± 0.00^b^
	2	0.31 ± 0.01^c^		2	0.69 ± 0.09^c^
	4	0.39 ± 0.00^d^		4	0.91 ± 0.11^d^
	8	0.59 ± 0.01^e^		8	1.41 ± 0.05^e^
BHT (mg/ml)	1	0.70 ± 0.01^f^	BHT (mg/ml)	1	0.70 ± 0.01^c^

Note

Values followed by different letters within the same columns are significantly different according to the Tukey's test (*p* ≤ .05).

#### 3.2.3 Antioxidant interactions between RES and EUG in CMC films

The RES and EUG concentrations which showed better antioxidant activities in CMC films based on DPPH and reducing power tests were incorporated into films in combination forms to evaluate their antioxidant interactions. For this purpose, concentrations of 4 and 8 µg/ml of RES as well as 4 and 8 mg/ml of EUG were added to films in combination forms. Table 3 shows the results of interaction antioxidant effects between RES and EUG in CMC films according to DPPH and reducing power assays. The C.I values of all combinational forms were higher than 1, which demonstrates the synergistic antioxidant effects between RES and EUG in CMC films, which means that simultaneous use of RES and EUG has a higher antioxidant activity compared with individual use of them. Furthermore, the film containing RES (8 µg/ml) + EUG (8 mg/ml) had the best synergistic antioxidant effects with 95.33% scavenging activity and 1.61 adsorption rate, as well as 1.09 and 1.92 C.I values, according to DPPH and reducing power tests, respectively (*p* ≤ .05). Skroza et al. (2015) studied the antioxidant activity of RES in combination with gallic acid, caffeic acid, and quercetin using the DPPH method and found that this phenolic mixture had an antagonistic effect in inhibiting DPPH free radicals, while the combination of RES and catechin indicated a synergistic effect (Skroza et al., 2015). In another study, the antioxidant interactions of RES and *Zataria multiflora* Boiss essential oil in sodium alginate film were evaluated and the synergistic effect was shown between them (C.I > 1) (Hashemi et al., 2019).

**TABLE 3:** Antioxidant interactions between RES and EUG in CMC films (Mean ± SD)

Mixture	DPPH (%)	C.I^a^	Reducing power (Absorbance at 700 nm)	C.I
RES (4 µg/ml) + EUG (4 mg/ml)	88.33 ± 1.15^b^	1.09	1.28 ± 0.06^b^	2.36
RES (8 µg/ml) + EUG (4 mg/ml)	92.33 ± 0.58^c^	1.09	1.37 ± 0.00^b^	1.9
RES (4 µg/ml) + EUG (8 mg/ml)	90.33 ± 0.58^bc^	1.08	1.41 ± 0.01^b^	2.33
RES (8 µg/ml) + EUG (8 mg/ml)	95.33 ± 1.53^d^	1.09	1.61 ± 0.08^c^	1.92

Note

Values followed by different letters within the same columns are significantly different according to the Tukey's test (*p* ≤ .05).

^a^ C.I: combination index. (S): Synergistic effect (C.I > 1); (Ad): Additive effect (C.I = 1); (A): Antagonistic effect (C.I < 1).

#### 3.2.4 Correlation between total phenolic content and antioxidant capacity

**FIGURE 2:**
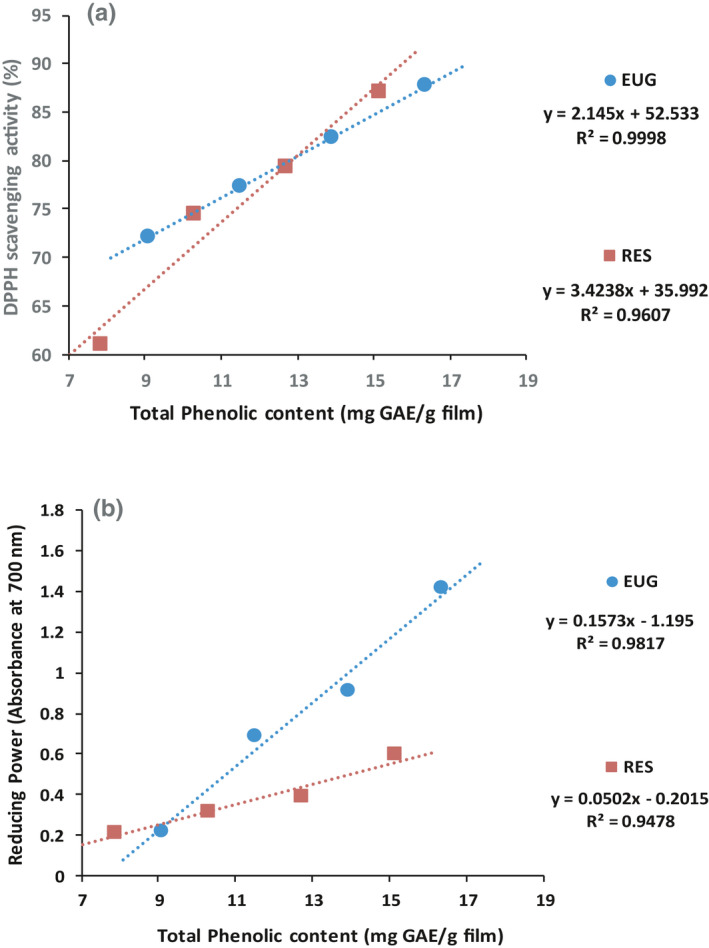
Correlation between total phenolic content and antioxidant capacity of CMC films incorporated with RES and EUG based on DPPH (a) and reducing power (b) tests

Phenolic compounds as antioxidant agents are able to trap free radicals, donate electrons or hydrogen atoms, and inactivate them (Hashemi et al., 2019; Zheng et al., 2019). Figure 2a,b represent correlation between total phenolic content and antioxidant capacity of CMC films containing RES or EUG according to DPPH and reducing power tests, respectively. The coefficient of determination (R^2^) for films containing RES or EUG based on DPPH test is 0.9607 and 0.9998, and based on reducing power, these numbers are 0.9478 and 0.9817, respectively.

The results of Pearson correlation analysis (*r*) between the total phenolic content and the antioxidant power of films containing RES or EUG based on DPPH test were 0.713 (significant at the 0.01 level) and 0.698 (significant at the 0.05 level), respectively. Also, according to reducing power tests, the results of mentioned analysis on the films containing RES or EUG were 0.773 (significant at the 0.01 level) and 0.750 (significant at the 0.01 level), respectively. The results of this analysis on the film containing RES (8 µg/ml) + EUG (8 mg/ml) (which demonstrated more favorable antioxidant effects among all combinational forms) were 0.581 (significant at the 0.05 level) and 0.707 (significant at the 0.05 level) according to the DPPH and reducing power tests, respectively. As a result, it can be concluded that the phenolic compounds in the films are responsible for their antioxidant activity. Several studies have been confirmed the correlation between the phenolic compounds and the antioxidant activity of active films (García et al., 2020; Kavoosi et al., 2014).

### 3.3 Antibacterial activities of CMC films containing RES and/or EUG

#### 3.3.1 Disk diffusion method

The results of antibacterial activities of CMC films containing RES and EUG using disk diffusion method are presented in Tables 4 and 5, respectively. By increasing the concentration of RES or EUG in CMC films, the antimicrobial effects of the films against *L. monocytogenes*, *S. aureus*, *S. enteritidis,* and *E. coli* were significantly increased (*p* ≤ .05). In addition, gram‐positive bacteria were more sensitive to the all treated films. The antibacterial activity of films containing RES was weak at all concentrations and against all bacteria. However, the antibacterial activities of films containing 1 and 2 mg/ml EUG as well as 4 and 8 mg/ml EUG against all studied bacteria were considered as weak and moderate, respectively. The films containing 8 μg/ml RES and 8 mg/ml EUG showed the highest antibacterial activities against *L. monocytogenes* with the inhibition zone diameters of 8.87 and 19.77 mm, respectively. Also, the lowest antimicrobial effects were recorded at concentrations of 1 μg/ml RES and 1 mg/ml EUG against *E. coli* with the inhibition zone diameters of 2.57 and 5.50 mm, respectively.

**TABLE 4:** Antibacterial activity of CMC films incorporated with RES using agar disk diffusion method (Mean ± SD)

Sample	Concentration	Inhibition zones (mm)			
		*L. monocytogenes*	*S. aureous*	*S. enteritidis*	*E. coli*
RES (µg/ml)	1	4.67 ± 0.35^b^ (+)	3.93 ± 0.51^b^ (+)	3.10 ± 0.53^b^ (+)	2.57 ± 0.50^b^ (+)
	2	6.47 ± 0.55^c^ (+)	5.33 ± 0.50^bc^ (+)	4.27 ± 0.60^bc^ (+)	3.77 ± 0.51^bc^ (+)
	4	7.73 ± 0.38^cd^ (+)	7.17 ± 0.64^cd^ (+)	5.80 ± 0.56^cd^ (+)	5.43 ± 0.78^cd^ (+)
	8	8.87 ± 0.50^d^ (+)	8.47 ± 0.59^d^ (+)	7.27 ± 0.55^d^ (+)	6.87 ± 0.65^d^ (+)
Chl^a^ (µg /disk)	30	29.03 ± 1.24^e^ (+++)	28.93 ± 1.30^e^ (+++)	26.23 ± 0.86^e^ (+++)	25.57 ± 1.12^e^ (+++)

Note

(+): Weak antibacterial activity (12 mm ≥ inhibition zone); (++): Moderate antibacterial activity (20 mm > inhibition zone > 12 mm); (+++): Strong antimicrobial activity (20 mm ≤ Inhibition zone).

Values followed by different letters within the same columns are significantly different according to the Tukey's test (*p* ≤.05).

^a^ Chl: Chloramphenicol (positive control).

**TABLE 5:** Antibacterial activity of CMC films incorporated with EUG using agar disk diffusion method (Mean ± SD)

Sample	Concentration	Inhibition zones (mm)			
		*L. monocytogenes*	*S. aureous*	*S. enteritidis*	*E. coli*
EUG (mg/ml)	1	7.13 ± 0.83^b^ (+)	6.63 ± 0.61^b^ (+)	5.73 ± 0.67^b^ (+)	5.50 ± 0.40^b^ (+)
	2	11.90 ± 0.85^c^ (+)	10.80 ± 1.39^c^ (+)	10.20 ± 1.21^c^ (+)	10.17 ± 1.11^c^ (+)
	4	15.83 ± 1.04^d^ (++)	14.67 ± 1.15^d^ (++)	13.83 ± 1.26^d^ (++)	13.67 ± 0.42^d^ (++)
	8	19.77 ± 0.84^e^ (++)	18.17 ± 1.26^e^ (++)	15.83 ± 1.18^d^ (++)	14.87 ± 1.47^d^ (++)
Chl^a^ (µg/disk)	30	29.03 ± 1.24^f^ (+++)	28.93 ± 1.30^f^ (+++)	26.23 ± 0.86^e^ (+++)	25.57 ± 1.12^e^ (+++)

Note

(+): Weak antibacterial activity (12 mm ≥ inhibition zone); (++): Moderate antibacterial activity (20 mm > inhibition zone > 12 mm); (+++): Strong antimicrobial activity (20 mm ≤ Inhibition zone).

Values followed by different letters within the same columns are significantly different according to the Tukey's test (*p* ≤ .05).

^a^ Chl: Chloramphenicol (positive control).

The researches have been proven that gram‐negative bacteria are more resistant to phenolic compounds than gram‐positives due to the limited release of hydrophobic compounds by their outer lipopolysaccharide membrane. In gram‐positive bacteria, the bilayer phospholipid membrane interacts with the hydrophobic elements in phenolic compounds, and consequently, cell death occurs due to increased ion permeability, leakage of vital cell compounds to the outside, and disruption of the bacterial enzyme system (Sandri et al., 2007). The higher susceptibility of gram‐positive bacteria to RES and EUG compared with gram‐negatives has been proven by another studies (Ma et al., 2013, 2018). In the study by Zheng et al. (2019), the antibacterial effects of biodegradable films containing EUG have been attributed to increasing non‐specific permeability of cytoplasmic membrane resulting in the disruption of the membrane (Zheng et al., 2019). However, in a study by Sanla‐Ead et al. (2012), the cellulose film containing EUG did not form any inhibition zone against any of the studied bacteria, due to the low solubility of EUG in antimicrobial films and the lack of emulsifiers. It has been proved that emulsifiers such as Tween 80 and Tween 20 can help antimicrobial agents to penetrate the bacterial cell membrane (Sanla‐Ead et al., 2012). Silva et al. (2016) reported significant antimicrobial activity of cellulose‐derived film containing RES against gram‐negative bacteria (Campylobacter and Arcobacter species) (Silva et al., 2016). In another study, the antibacterial activity of chitosan–starch film containing RES was confirmed by disk diffusion method (Lozano‐Navarro et al., 2017). The antibacterial activity of RES against foodborne pathogens has been attributed to several mechanisms including DNA degradation, cell division impairment, membrane oxidative damage, and inhibition of enzymes involved in the electron transport chain. Gram‐negative bacteria were more resistant to RES due to the presence of an external hydrophilic membrane. The outer hydrophilic membrane can be considered as a protective layer against hydrophobic compounds such as RES. The presence of degradative and detoxifying enzymes in the periplasmic space of gram‐negative bacteria could be another contributing factor to reduced activity of RES due to breakdown of the RES molecule by these enzymes. Also, multidrug‐resistant pumps on the surface of gram‐negative bacteria may be able to extrude RES from the cell (Ma et al., 2018).

#### 3.3.2 Broth dilution method

The results of antibacterial effects of CMC films containing RES using broth dilution method are shown in Table 6. Comparison of the results of different treatments with the 24‐h control group shows that the addition of 2, 4, and 8 μg/ml RES to CMC films against *L. monocytogenes*, *S. aureus,* and *S. enteritidis* and addition of 4 and 8 μg/ml RES to CMC films against *E. coli* significantly reduced the population of bacteria (*p* ≤ .05). The antibacterial capacity of the films was significantly increased with increasing the RES concentration (*p* ≤ .05). Gram‐positive bacteria were also more sensitive to films containing RES. Consequently, the highest antibacterial effect between films containing RES was related to the film containing 8 μg/ml RES against *L. monocytogenes* with the percentage of bacterial growth inhibition (P.I) of 12.39%. Also, the lowest antibacterial effects were recorded for the film containing 1 μg/ml RES against *E. coli* with a P.I value of 0.14%.

**TABLE 6:** Antibacterial activity of CMC films incorporated with RES using broth dilution method (Mean ± SD)

Sample	Concentration (µg/ml)	*L. monocytogenes*			*S. aureous*			*S. enteritidis*			*E. coli*		
		B.C*	B.R**	P.I***	B.C	B.R	P.I	B.C	B.R	P.I	B.C	B.R	P.I
Control	After 0 h	5.18 ± 0.20^a^	^—^	^—^	5.23 ± 0.14^a^	^—^	^—^	5.22 ± 0.09^a^	^—^	^—^	5.25 ± 0.10^a^	^—^	^—^
	After 24 h	6.17 ± 0.07^d^	^—^	^—^	6.20 ± 0.09^d^	^—^	^—^	6.20 ± 0.07^d^	^—^	^—^	6.19 ± 0.12^c^	^—^	^—^
RES	1	5.96 ± 0.08^cd^	0.21 (‐)	3.44	6.04 ± 0.14^cd^	0.15 (‐)	2.51	6.10 ± 0.15^cd^	0.09 (‐)	1.65	6.18 ± 0.09^c^	0.01 (‐)	0.14
	2	5.81 ± 0.16^c^	0.35 (‐)	5.81	5.86 ± 0.09^c^	0.33 (‐)	5.38	5.91 ± 0.09^bc^	0.29 (‐)	4.71	5.97 ± .0.11^bc^	0.22 (‐)	3.55
	4	5.67 ± 0.11^bc^	0.49 (‐)	8.09	5.73 ± 0.12^bc^	0.46 (‐)	7.49	5.80 ± 0.10^b^	0.40 (‐)	6.52	5.86 ± 0.07^b^	0.34 (‐)	5.35
	8	5.41 ± 0.06^ab^	0.77 (+)	12.39	5.54 ± 0.07^ab^	0.66 (+)	10.67	5.70 ± 0.06^b^	0.50 (+)	7.98	5.78 ± 0.06^b^	0.42 (‐)	6.59

Note

Values followed by different letters within the same columns are significantly different according to the Tukey's test (*p* ≤.05).

* Bacterial counts [log (CFU/ml)].

** Bacterial reduction index. (‐): Nonsignificant antibacterial activity (0.5 > B.R); (+): Slight antibacterial activity (1 > B.R ≥ 0.5); (++): Significant antibacterial activity (3 > B.R ≥ 1); (+++): Strong antibacterial activity (3 ≤ B.R).

*** Percentage of inhibition (%).

According to the results of the bacterial reduction index (B.R), the order of susceptibility of the studied bacteria to the CMC films containing RES was as follows: *L*. *monocytogenes* > *S*. *aureus* > *S*. *enteritidis* > *E*. *coli*. Accordingly, the highest antibacterial effect belonged to the CMC film containing 8 μg/ml RES against *L. monocytogenes* with a B.R index of 0.77 (slight antibacterial activity) and the lowest antibacterial effect belonged to the CMC film containing 1 μg/ml RES against *E. coli* with a B.R index of 0.01 (nonsignificant antibacterial activity).

The results of antibacterial activities for CMC films containing EUG using broth dilution method can be observed in Table 7. Results indicated that the addition of 2, 4, and 8 mg/ml EUG to the CMC edible films significantly decreased the population of *L. monocytogenes*, *S. aureus,* and *S. enteritidis* compared with 24‐h control group (*p* ≤ .05). But, antibacterial activity of the films against *E. coli* was just significant at the concentration 4 and 8 mg/ml EUG (*p* ≤ .05). The inhibitory effects of the films also increased significantly with increasing EUG concentration (*p* ≤ .05). Additionally, gram‐positive bacteria showed higher sensitivity to the films containing EUG and the highest antibacterial activity belonged to the film containing 8 mg/ml EUG against *L. monocytogenes* with P.I value of 24.09%. Also, the lowest antibacterial activity was recorded with the film containing 1 mg/ml EUG on *E. coli* with P.I value of 0.65%.

**TABLE 7:** Antibacterial activity of CMC films incorporated with EUG using broth dilution method (Mean ± SD)

Sample	Concentration (mg/ml)	*L. monocytogenes*			*S. aureous*			*S. enteritidis*			*E. coli*		
		B.C*	B.R**	P.I***	B.C	B.R	P.I	B.C	B.R	P.I	B.C	B.R	P.I
Control	After 0 h	5.18 ± 0.20^b^	^—^	^—^	5.23 ± 0.14^b^	^—^	^—^	5.22 ± 0.09^a^	^—^	^—^	5.25 ± 0.10^a^	^—^	^—^
	After 24 h	6.17 ± 0.07^d^	^—^	^—^	6.20 ± 0.09^d^	^—^	^—^	6.20 ± 0.07^d^	^—^	^—^	6.19 ± 0.12^c^	^—^	^—^
EUG	1	5.82 ± 0.19^cd^	0.33 (‐)	5.76	5.91 ± 0.10^cd^	0.28 (‐)	4.58	6.03 ± 0.12^cd^	0.16 (‐)	2.73	6.15 ± 0.10^c^	0.04 (‐)	0.65
	2	5.52 ± 0.15^bc^	0.64 (+)	10.58	5.64 ± 0.15^c^	0.55 (+)	9.01	5.85 ± 0.13^bc^	0.34 (‐)	5.66	5.97 ± 0.08^bc^	0.23 (‐)	3.66
	4	5.18 ± 0.12^b^	0.99 (+)	16.14	5.31 ± 0.08^b^	0.89 (+)	14.28	5.68 ± 0.04^b^	0.52 (+)	8.34	5.79 ± 0.09^b^	0.41 (‐)	6.50
	8	4.68 ± 0.08^a^	1.49 (++)	24.09	4.82 ± 0.09^a^	1.38 (++)	22.19	5.24 ± 0.13^a^	0.95 (+)	15.45	5.33 ± 0.10^a^	0.87 (+)	13.94

Note

Values followed by different letters within the same columns are significantly different according to the Tukey's test (*p* ≤.05).

* Bacterial counts [log (CFU/ml)]

** Bacterial reduction index. (‐): Nonsignificant antibacterial activity (0.5 > B.R); (+): Slight antibacterial activity (1 > B.R ≥ 0.5); (++): Significant antibacterial activity (3 > B.R ≥ 1); (+++): Strong antibacterial activity (3 ≤ B.R).

*** Percentage of inhibition (%).

According to the results of B.R index, the order of susceptibility of the studied bacteria to the CMC films containing EUG was as follows: *L*. *monocytogenes* > *S*. *aureus* > *S*. *enteritidis* > *E*. *coli*. The sensitivity of gram‐positive bacteria is more than gram‐negatives. Accordingly, the highest antibacterial activity belonged to the CMC film containing 8 mg/ml EUG against *L. monocytogenes* with a B.R index of 1.49 (significant antibacterial activity). However, the lowest antibacterial effect recorded for the film containing 1 mg/ml EUG against *E. coli* with a B.R index of 0.04 (nonsignificant antibacterial activity).

#### 3.3.3 Antibacterial interactions between RES and EUG in CMC films

The results of antibacterial interactions between RES and EUG in CMC films using disk diffusion method are presented in Table 8. In general, the inhibitory zones of combined treatments were higher than single treatments and films containing 8 mg/ml EUG along with different concentrations of RES demonstrated strong antimicrobial activity against gram‐positive bacteria (Inhibition zone ≥ 20 mm). However, these results demonstrate the antagonistic effects of the combined use of RES and EUG compared with their alone use in the films. In other words, the "expected inhibition zones" were smaller than the inhibition zones of CMC films containing a combination of RES and EUG. Also, the inhibitory effects of the films containing RES (8 µg/ml) + EUG (8 mg/ml) and RES (4 µg/ml) + EUG (8 mg/ml) against all studied bacteria were significantly more than other combined treatments (*p* ≤ .05).

**TABLE 8:** Antibacterial interactions between RES and EUG in CMC films using agar disk diffusion method (Mean ± SD)

Bacteria	Inhibition Zones (mm)							
	Expected inhibition zones				Occurred inhibition zones			
	RES (4 µg/ml) + EUG (4 mg/ml)	RES (8 µg/ml) + EUG (4 mg/ml)	RES (4 µg/ml) + EUG (8 mg/ml)	RES (8 µg/ml) + EUG (8 mg/ml)	RES (4 µg/ml) + EUG (4 mg/ml)	RES (8 µg/ml) + EUG (4 mg/ml)	RES (4 µg/ml) + EUG (8 mg/ml)	RES (8 µg/ml) + EUG (8 mg/ml)
*L. monocytogenes*	23.56	24.70	27.50	28.33	18.73 ± 0.46^a^ (A) (++)	19.07 ± 0.42^a^ (A) (++)	21.53 ± 0.35^b^ (A) (+++)	22.10 ± 0.40^b^ (A)(+++)
*S. aureous*	21.83	23.13	25.33	26.63	18.17 ± 0.45^a^ (A) (++)	18.77 ± 0.60^a^ (A) (++)	20.53 ± 0.42^b^ (A) (+++)	21.47 ± 0.42^b^ (A) (+++)
*S. enteritidis*	19.63	21.10	21.63	23.10	16.07 ± 0.42^a^ (A) (++)	16.40 ± 0.66^a^ (A) (++)	17.63 ± 0.35^b^ (A) (++)	18.73 ± 0.32^b^ (A) (++)
*E. coli*	19.10	20.53	20.30	21.73	15.37 ± 0.51^a^ (A) (++)	16.07 ± 0.42^a^ (A) (++)	17.40 ± 0.26^b^ (A) (++)	18.07 ± 0.32^b^ (A) (++)

Note

(S): Synergistic effect; (Ad): Additive effect; (A): Antagonistic effect.

(+): Weak antibacterial activity (12 mm ≥ inhibition zone); (++): Moderate antibacterial activity (20 mm > inhibition zone > 12 mm); (+++): Strong antimicrobial activity (20 mm ≤ Inhibition zone).

Values followed by different letters within the same rows are significantly different according to the Tukey's test (*p* ≤ .05).

The results of antibacterial interactions between RES and EUG in CMC films using broth dilution method are presented in Table 9. In general, the antibacterial effects of combined treatments were higher than single use of RES or EUG in CMC films. Comparing the results of various combined treatments with the control group showed the significant reduction of *L. monocytogenes*, *S. aureus*, *S. enteritidis,* and *E. coli* counts (*p* ≤ .05). Also, all of the combined treatments had a B.R index of more than 0.5 (slight and significant antibacterial activities). The highest antibacterial effects were related to the film containing RES (8 µg/ml) + EUG (8 mg/ml) against *L. monocytogenes* with the P.I value and B.R index of 29.43% and 1.82 (significant antibacterial effect), respectively. In addition, the lowest antimicrobial effects were recorded for the film containing RES (4 µg/ml) + EUG (4 mg/ml) against *E. coli* with the P.I value and B.R index of 9.31% and 0.58 (slight antibacterial effect), respectively.

**TABLE 9:** Antibacterial interactions between RES and EUG in CMC films using broth dilution method (Mean ± SD)

Sample	*L. monocytogenes*			*S. aureous*			*S. enteritidis*			*E. coli*		
	B.C*	B.R**	P.I***	B.C	B.R	P.I	B.C	B.R	P.I	B.C	B.R	P.I
Control (after 0 h)	5.18 ± 0.20^c^	^—^	^—^	5.23 ± 0.14^b^	^—^	^—^	5.22 ± 0.09^bc^	^—^	^—^	5.25 ± 0.10^bc^	^—^	^—^
Control (after 24 h)	6.17 ± 0.07^d^	^—^	^—^	6.20 ± 0.09^c^	^—^	^—^	6.20 ± 0.07^e^	^—^	^—^	6.19 ± 0.12^e^	^—^	^—^
RES (4 µg/ml) + EUG (4 mg/ml)	5.03 ± 0.08^bc^	1.14 (++)	18.53	5.13 ± 0.06^b^	1.07 (++)	17.20	5.51 ± 0.04^d^	0.69 (+)	11.15	5.62 ± 0.07^d^	0.58 (+)	9.31
RES (8 µg/ml) + EUG (4 mg/ml)	4.81 ± 0.08^b^	1.36 (++)	22.03	5.03 ± 0.04^b^	1.17 (++)	18.87	5.35 ± 0.05^cd^	0.85 (+)	13.76	5.43 ± 0.09^cd^	0.76 (+)	12.27
RES (4 µg/ml) + EUG (8 mg/ml)	4.51 ± 0.05^a^	1.66 (++)	26.89	4.64 ± 0.10^a^	1.55 (++)	25.05	5.06 ± 0.08^b^	1.14 (++)	18.40	5.11 ± 0.07^ab^	1.09 (++)	17.46
RES (8 µg/ml) + EUG (8 mg/ml)	4.36 ± 0.07^a^	1.82 (++)	29.043	4.52 ± 0.03^a^	1.69 (++)	27.11	4.84 ± 0.10^a^	1.36 (++)	22.00	4.95 ± 0.03^a^	1.25 (++)	20.09

Note

Values followed by different letters within the same columns are significantly different according to the Tukey's test (*p* ≤ .05).

* Bacterial counts [log (CFU/ml)].

** Bacterial reduction index. (‐): Nonsignificant antibacterial activity (0.5 > B.R); (+): Slight antibacterial activity (1 > B.R ≥ 0.5); (++): Significant antibacterial activity (3 > B.R ≥ 1); (+++): Strong antibacterial activity (3 ≤ B.R).

*** Percentage of bacterial growth inhibition (%).

Ma et al. (2013) reported the antagonistic effects of the combined use of EUG and lauric arginate on gram‐negative bacteria (*E. coli* and *S. enteritidis*) and their synergistic effects on gram‐positive bacteria (*L. monocytogenes*). The mentioned study attributed the mechanism of antagonistic effects against gram‐negative bacteria to the initial binding of lauric arginate to the lipopolysaccharide membrane and the increase in membrane thickness and resistance to EUG (Ma et al., 2013). Tosato et al. (2018) demonstrated the antagonistic effects of RES on levofloxacin antibiotic. They reported that RES eliminates reactive oxygen species due to its inherent antioxidant effects and produces antagonistic effects. Reactive oxygen species are produced during some antimicrobial therapies, including antibiotic therapy, and cause the death of microorganisms (Tosato et al., 2018). However, synergistic (ciprofloxacin) and additive (cefotaxime) effects between RES and antibiotics have also been reported against gram‐positive and gram‐negative bacteria (Kumar et al., 2012). In general, some antimicrobial interaction mechanisms have been accepted regarding antimicrobial synergistic effects: sequential inhibition of a common biochemical pathway, inhibition of protective enzymes, combinations of cell wall‐active agents, and use of cell wall‐active agents to enhance the uptake of other antimicrobials (Santiesteban‐López et al., 2007). The possible disruption of one or more of these mechanisms increases the possibility of antagonistic effects.

## 4 CONCLUSION

The use of RES and EUG to the edible CMC films increased the total phenolic content and increased their antioxidant and antibacterial activities against gram‐positive bacteria (*L*. *monocytogenes* and *S*. *aureus*) and gram‐negative bacteria (S. *enteritidis* and *E*. *coli*). Antioxidant and antimicrobial properties of the CMC films increased with increasing concentration of phenolic compounds. Gram‐positive bacteria were more sensitive than gram‐negatives, and the highest antibacterial activity was observed against *L. monocytogenes*. The combined use of RES and EUG in CMC films produced the synergistic antioxidant effects. However, despite the stronger antibacterial effects of combined use of RES and EUG than single use of each of them in the film, the combined use of them produced the antagonistic antibacterial effects. The best results were related to the CMC film containing RES (8 µg/ml) + EUG (8 mg/ml). Therefore, based on the findings of the present study, the edible CMC films containing RES and EUG can be used for the food packaging industry.

## CONFLICT OF INTEREST

Authors declare no conflict of interest.

## AUTHOR CONTRIBUTION

**Majid Aminzare:** Conceptualization (lead); Data curation (equal); Formal analysis (lead); Funding acquisition (lead); Investigation (equal); Methodology (lead); Project administration (lead); Resources (lead); Software (equal); Supervision (lead); Validation (equal); Visualization (equal); Writing‐original draft (equal); Writing‐review & editing (lead). **Roya Moniri:** Data curation (equal); Formal analysis (equal); Investigation (equal); Validation (equal); Visualization (equal); Writing‐original draft (equal); Writing‐review & editing (equal). **hassan hassanzadazar:** Conceptualization (supporting); Funding acquisition (supporting); Investigation (supporting); Methodology (supporting); Project administration (supporting); Resources (supporting); Supervision (supporting); Validation (supporting); Visualization (supporting); Writing‐original draft (supporting); Writing‐review & editing (supporting). **Mohammad Reza Mehrasbi:** Conceptualization (supporting); Data curation (supporting); Formal analysis (supporting); Funding acquisition (supporting); Investigation (supporting); Methodology (supporting); Project administration (supporting); Resources (supporting); Software (supporting); Supervision (supporting); Validation (supporting); Visualization (supporting); Writing‐original draft (supporting); Writing‐review & editing (supporting).

## Data Availability

The data that support the findings of this study are openly available in “figshare” at https://figshare.com/articles/dataset/CMC_films_containing_RES_and_EUG_xlsx/15062280.
